# “Yes, we know!” (Over)confidence in general knowledge among Austrian entrepreneurs

**DOI:** 10.1371/journal.pone.0197085

**Published:** 2018-05-08

**Authors:** Viktorija Ilieva, Thomas Brudermann, Ljubomir Drakulevski

**Affiliations:** 1 Faculty of Economics, Ss. Cyril and Methodius University in Skopje, Skopje, Macedonia; 2 Institute of Systems Sciences, Innovation and Sustainability Research, University of Graz, Graz, Austria; Medical University Graz, AUSTRIA

## Abstract

Overconfidence has been reported to be a common bias among entrepreneurs and might be one cause of the high failure rates observed for new ventures. In this study, we investigate the overconfidence bias in a sample of 92 Austrian entrepreneurs, who responded to a general-knowledge questionnaire. Their levels of overconfidence were assessed by their responses to hard, medium and easy knowledge questions, and the relations of individual, organizational and environmental factors to the bias score were analyzed. The results confirmed that entrepreneurs are indeed prone to expressing overprecision, a type of overconfidence, but not when answering questions of all levels of difficulty. Being a single founder instead of a co-founder was identified as a significant predictor of overconfidence. Confidence, on the other hand, was associated with age and prior entrepreneurial experience, while accuracy was determined solely by age. The results of this study only partly agree with those of previous studies conducted in different national and cultural settings.

## 1. Introduction

### 1.1. Entrepreneurial miscalibration

Understanding how founders of new ventures assess probability or make judgments under conditions of uncertainty is essential if we define the entrepreneur as someone who specializes in taking responsibility for and making judgmental decisions under such conditions [1,2]. While the market entry may be attributed to optimism [3] or overplacement, in terms of confidence in one’s skill relative to that of others [4], market success could be predicted by accuracy of calibration. There is a positive relationship between entrepreneurial success and appropriate calibration of the entrepreneurs (Bonnefon et al. 2006, reported in [5]. Miscalibrated entrepreneurs achieved worse results with regard to venture performance and individual rewards [6]. Accuracy in judgment can produce more positive outcomes in competitive market situations compared to motivation and persistence [7].

Overconfidence has been reported to be one prevalent, intrinsic, cognitive bias expressed by entrepreneurs and has been shown to have detrimental effects on decision outcomes [8]. But as more research on this cognitive bias is conducted, it becomes more important to become aware of the different definitions and measures applied in various studies in the field when overconfidence is addressed. These variations in definitions are a great obstacle toward understanding overconfidence [9] in general and as a cognitive bias germane to entrepreneurs. In this regard, [10] differentiated among three types of overconfidence: overestimation, overplacement and overprecision. Pertaining to entrepreneurs, the dominant biases that have been studied as a form of overconfidence are overestimation, overoptimism and overprecision.

Overestimation–defined as the overconfidence in the ability to do something–has been the basis of a great deal of research in entrepreneurship and is the most commonly studied form of overconfidence [8]. In this sense, overestimation refers to: an event having a positive outcome [11], the skills, knowledge and ability needed to start a business [12] and the predictive validity of a cue [13]. The way overestimation is measured also varies among researchers: e.g., the difference between what was estimated in the budget and the actual results [11], differences between self-reports of life expectancy and the actual life span [14], or simply using the level of effort of the entrepreneur as a proxy for overconfidence [15].

Overoptimism is defined as the tendency towards believing and reporting to be more likely than others to gain positive outcomes [16–19]; or being certain of success and facing disappointment at the end [20]. Some of the ways to measure overoptimism include asking respondents if the outcomes of a new-venture creation are in accordance with what they expected at the beginning [19] or, with reference to comparative overoptimism, asking them to rank the odds that they will achieve success in their business as well as for any other business like theirs [18].

Overprecision is another judgmental bias which is not well understood [21]. In the literature, overprecision is also found as one type of overconfidence, and some authors refer to it as judgmental overconfidence [17]. Overprecision is the excessive, expressed belief that one knows the truth [22] or the overestimation of the precision of one’s knowledge or information [3,17,23,24]. The findings of the experiment conducted by [17] indicated that judgmental overconfidence inhibits innovation.

### 1.2. Determinants of overconfidence

When dealing with overconfidence in terms of general knowledge, the hard-easy effect should be acknowledged. This effect is related to the ubiquitous occurrences in which people appear more overconfident when faced with hard questions or tasks, while their overconfidence decreases when they are faced with easier items or tasks [2,24]. The moderating role of the level of difficulty of the questions or tasks on overconfidence [4] was framed in entrepreneurial terms by [25] as: “overconfidence is particularly likely when tasks are difficult and/or when judgments are made with high confidence, conditions that characterize those which founders face”.

Overprecision is a specific type of overconfidence, and factors that determine other types of overconfidence may not be (the only ones) applicable to overprecision. An argument that supports this assumption appears in the findings of research comparing general-knowledge overconfidence to other affiliated biases such as overoptimism. The findings of this research showed that each of these is a distinct bias with distinct effects [17,20]. And last but not least, determinants that were shown to be explanatory in samples of students may not be explanatory when entrepreneurs are examined. Therefore, when interpreting the findings of this and other studies on overprecision, the definitions used, methodology applied to measure overconfidence and the sample used must be considered while drawing comparisons with the findings of other studies on determinants of entrepreneurial overconfidence.

Factors that have been most often linked to entrepreneurial overconfidence, irrespective of its form, include: age [6,26,27], education [11,12,19], entrepreneurial experience [12,18,25], firm age [27], ownership structure [11,19], external investments [27,28], innovative activity [13,17,20,29] and environmental dynamism [6,19,20,25].

### 1.3. Aims of the paper

Overconfidence in general knowledge has not been studied extensively among European entrepreneurs, to the best of our knowledge. The sample of [28] included 764 inventor-entrepreneurs from Canada; [6] used a sample of 102 entrepreneurs from the USA; Busenitz and Barney’s [26] sample included 124 entrepreneurs from the USA; and [27] gathered responses from 97 entrepreneurs in the USA. Herz et al.’s [17] sample is unique as it consisted of 119 Swiss students and 35 Swiss managers. We assumed that European/Austrian entrepreneurs do not significantly differ from North-American entrepreneurs with regards to overconfidence, considering the global culture of entrepreneurship [30], and we expected that entrepreneurs from the Austrian sample would manifest overconfidence as well. The hypotheses regarding determinants of entrepreneurial overprecision, therefore, were developed on the basis of insights gained from the reviewed international studies.

## 2 Methods

### 2.1. Development of hypotheses

This study was conducted to examine overconfidence among Austrian entrepreneurs. Based on possible determinants of overconfidence found in the peer-reviewed scientific literature, we formulated the following hypotheses.

*H1*: *Entrepreneurial overprecision decreases with age*.

Age plays an important role in entrepreneurial decision-making, and cognitive processes change as people get older [31]. In general, the probability of starting a business has been found to be higher among older individuals but only up to a certain age, at which this probability starts to decrease [32].

The findings of [6,27] indicated that younger entrepreneurs are more overconfident than older ones. By observing the effect of age on overestimation in the entrepreneurial forecasts of financial data, [11] found no significant effect unless the entrepreneur was younger than 35 years of age. According to [33], older managers usually search for more information before making a decision, and they are generally less confident about the decision reached.

*H2*: *Higher levels of education negatively affect the level of entrepreneurial overprecision*.

Education seems to be a factor that simply cannot be neglected when discussing overconfidence measured as overprecision. [11] concluded that education has a negative impact on overconfidence (measured as overestimation), and [2] reached the conclusion that “those who know more do not generally know more about how much they know” (p. 179) and, therefore, are even prone to underconfidence. [19] took a distinct approach when describing the role of education in entrepreneurial calibration: they differentiated between a general knowledge which positively relates to overoptimism and specific knowledge which leads to a reduction in overoptimism.

*H3*: *Prior entrepreneurial experience positively affects entrepreneurial overconfidence*.

Confidence and experience are associated, and positive feedback increases confidence while negative feedback decreases it [24].

The environment in which entrepreneurs work is dominated by information overload as well as high levels of uncertainty, novelty, emotion and time pressure which may lead to overconfident individuals [34]. In the model of [25], entrepreneurs were shown to be more prone to overconfidence when their current business was not similar to any prior entrepreneurial experience they had.

Nevertheless, evidence that experience has the opposite effect on overconfidence has also been found. Nascent entrepreneurs are more confident in terms of their expression of their skills, knowledge and experience than established ones [12]. But repeat entrepreneurs with no experience with business failure are more susceptible to comparative optimism than novice entrepreneurs [18].

*H4*: *Entrepreneurs running their business alone are more prone to overconfidence than those who are cofounders*.

While there has not been any research in terms of the overprecision and the ownership structure, our expectations can be based on the research focused on another type of overconfidence. Entrepreneurs running their businesses alone are more likely to overestimate forecasts for certain financial results, but entrepreneurial teams are also not immune to biased decision-making [11]. Regarding overoptimism, asking for outside help and not preferring to do everything alone have negative impacts [19].

*H5*: *Overprecision is positively related to attracting external investments*.

A well-established belief that appears in the entrepreneurial literature is that overconfidence might be beneficial to entrepreneurs during their negotiations with potential external investors. The use of external investments is viewed in two different ways regarding overconfidence. Firstly, entrepreneurial cognitive biases (planning fallacy in particular) have a positive impact on attracting investors. The more biased the entrepreneur (in terms of planning fallacy and optimism), the more likely they are to get loans from strong-tie investors (family and friends). The effect of these cognitive biases is the opposite when it comes to obtaining funding from weak-tie investors [28]. From another point of view, entrepreneurs who use external investments are less overconfident than those who do not [27], and this implies that the use of external investments “pushes” entrepreneurs to analyze information more carefully because they are held accountable by the investors.

*H6*: *There is a positive correlation between entrepreneurial overprecision and innovative activity*.

Judgmental overconfidence was observed to have a negative effect on innovative activity in an experimental task where the participants were students and business managers [17]. On the other hand, the findings of a study conducted with high-tech companies showed that overestimation was positively related to the introduction of more pioneering products rather than incremental products [13]. Overoptimism of product success has also been shown to be positively related to the introduction of more pioneering products that require more resources [20]. Using GEM data, [29] reported that innovation would more probably be found among individuals with higher levels of self-confidence.

*H7*: *The greater the environmental dynamism, the greater the entrepreneurial miscalibration*.

[27] referenced the study of environmental dynamism in relation to entrepreneurial overconfidence as a research gap, and [6] addressed this question, finding that overprecision increases with environmental dynamics. Optimistic overconfidence, on the other hand, has been negatively related to environmental dynamism [20]. Environmental hostility was observed separately in their study, and it increased optimistic overconfidence, while in the study of [6], environmental hostility appeared as one of the items that was incorporated in the measure of environmental dynamism.

By and large, our research adds to the existing literature on entrepreneurial overconfidence. We start by questioning whether Austrian entrepreneurs are miscalibrated, as has been suggested by the results of previous research. This research has mainly been conducted with American entrepreneurs. In contrast to previous studies, we apply an overprecision measure that deals with the hard-easy effect. First and foremost, we asked whether the individual, organizational and environmental factors examined in previous studies (target groups: American entrepreneurs or students), for this or other forms of overconfidence, also influence Austrian entrepreneurs’ levels of overprecision. To gain a better understanding of the entrepreneurial overconfidence in terms of knowledge, we also examined which factors are related to the two variables from which overconfidence is derived: the average confidence level and the accuracy.

### 2.2. Sample and data collection

The hypotheses were rigorously tested with data collected from a sample of ninety-two entrepreneurs from Austria. The data was collected in November and December 2017 by means of an online survey. An invitation letter, which included background information on the study and the link to the survey, was forwarded to entrepreneurs with the help of incubators, accelerators and other Austrian entrepreneurial networks. A reminder was sent after two weeks. For the purposes of this study, entrepreneurs were defined as individuals who had already started a business or were preparing to do so at the time of the study.

### 2.3. Test instrument / questionnaire

The questionnaire consisted of two parts. The first part contained questions on the individual, organizational and environmental factors that previous research had found to be related to entrepreneurial overconfidence. The second part of the survey was a general-knowledge questionnaire made up of 18 questions. The respondents were clearly informed about the anonymity of the submitted responses, and they were not told about the particular aspect of cognitive bias, the assessment of which was the goal of the research.

Overprecision is usually measured by using one of the following two approaches: setting confidence intervals in confidence-range tasks [35] or stating a confidence level for *n*-choice tasks [2,36]. In our study, we used the second way to measure overprecision, which is regarded as closer to the way people think about and express their confidence in everyday situations [37]. This approach requires the respondents to select the correct answer from *n* alternatives and, after that, to assign a confidence level that the provided answer is correct on a scale of 1/n*100 to 100 percent. The individual bias score (overconfidence level) was calculated as the difference between the average confidence level (certainty or knowledge perception) and the percentage of correct answers (accuracy or knowledge) given by each of the entrepreneurs. If the average confidence level is higher than the percentage of correct answers (or the bias score is positive), the person is considered to be overconfident; if it is lower (or the bias score is negative), they are considered to be underconfident; and if the bias score equals 0, the person is considered to be well-calibrated.

The method of measuring overprecision by asking general-knowledge questions has already been used in the entrepreneurial context [6,26,27], but those questionnaires only consisted of a few questions and were mainly used to identify the differences between entrepreneurs and managers regarding overconfidence. The hard-easy effect was neglected, because those questions represented moderate to high levels of difficulty [27].

[23,38] developed the *test-18* as an instrument to measure overprecision. Their instrument has not been used so far to study entrepreneurs. This study applies 16 questions from the original questionnaire *test-18*, while two questions were replaced with similar ones. Since the respondents were offered three possible answers from which to choose, they were instructed to assign the confidence level by using a scale of 33% (guessing) to 100% (completely sure). The resulting bias scores had a potential range of -67 (least overconfident) to 100 (most overconfident).

After obtaining the answers, the questions could be categorized into easy, medium and hard questions, based on the rate of correct answers. The goals here were to obtain three levels of difficulty with high degrees of homogeneity within each level, and heterogeneity between the three levels; and an equal number of questions in each difficulty level. Consequently, questions with 0–40% correct answers were classified as hard, questions with 40–70% correct answers as medium, and questions with 70–100% correct answers as easy, similar to the categorization made by [24].

Entrepreneurial age was coded in 28 categories, from 18 to 71 or older. The level of education was coded as a dichotomous variable: compulsory education/high school/Bachelor’s and Master’s/Doctoral degree. Prior entrepreneurial experience was coded as a dummy variable, dividing entrepreneurs into two groups: those with no prior experience (their current business is the only one they have ever been involved in) and those who had founded at least one venture prior to their current one. Ownership structure and external investments were coded as dummy variables. Innovative activity was coded as a dummy variable, derived from the answers to three questions. More details on the definition of the types of innovative activity are provided in [29]. Environmental dynamism was treated as a continuous variable and measured by asking five Likert-scale questions regarding the competitive environment, as applied in [6].

The questionnaire and collected survey data are provided in the S1 and S2 Files.

### 2.4. Ethics statement

As the survey did not involve the release of private/personal data, it was not necessary to gain the approval of the ethics committees of University of Graz and Ss. Cyril and Methodius University in Skopje. All data collected during this study were treated confidentially, in accordance with ethical standards and anonymized for the purpose of the analysis.

## 3. Results

Table 1 summarizes the descriptive statistics for the dependent variables confidence, accuracy and overconfidence. Subjects in the sample were on average overconfident (mean = 13.63, SD = 16.24, range: from -29.58 to 66.67; 15.2% were underconfident, 1.1% were well-calibrated and 83.7% were overconfident). The average confidence level measured in the sample was 63.72%, the average accuracy was 50.09% and the average overconfidence level was 13.63%. The entrepreneurs were not perfectly calibrated: When stating 100% confidence on the correctnes of an answer, the respondents only were correct in 71.6% of the cases.

**Table 1:** Descriptive statistics for the dependent variables.

	All questions		Easy		Medium		Hard	
	Mean	SD	Mean	SD	Mean	SD	Mean	SD
**Confidence**	63.72	12.04	73.23	15.72	48.87	14.48	69.07	14.06
**Accuracy**	50.09	16.23	80.98	24.69	49.46	27.48	19.84	19.45
**Overconfidence**	13.63	16.24	-7.75	19.91	-0.59	26.95	49.23	24.97

We observed a significant, moderately positive correlation between the accuracy and confidence scores (Pearson’s r = 0.37, *p* = 0.000) and between confidence and overconfidence (Pearson’s r = 0.37, *p* = 0.000).

Our results suggest that entrepreneurs displayed overconfidence when answering hard questions and underconfidence when answering easy questions or those of medium difficulty. Ninety out of the ninety-two entrepreneurs showed higher overconfidence scores for hard questions as compared to overconfidence they showed for easy questions, and only two were more overconfident when answering easy questions than when answering hard questions. A Wilcoxon signed-rank test of the data showed a significant increase in the overconfidence score when respondents answered the hard questions (Mdn = 49.75) compared to the overconfidence score obtained when they answered the easy questions (Mdn = -11.88), *z* = 8.290, *p* = 0.000.

Table 2 shows the results of the independent-samples t-tests which were used to determine if a difference exists between the means of two independent groups on the continuous dependent variables: overconfidence, confidence and accuracy. The Pearson's correlation coefficients that measure the statistical association between the continuous independent variables (age and environmental dynamism) and the continuous dependent variables, are shown in Table 3. Table 4 shows the results of the multiple regression analysis. The explanatory power of the models is rather low, as is typical when studying human behavior, and more particularly when studying overprecision or overestimation [11,27].

**Table 2:** Bivariate analysis for independent dummy variables and dependent variables.

		Overconfidence		Confidence		Accuracy	
	Percentage	Mean	SD	Mean	SD	Mean	SD
**Education**							
1 = with Master’s or Doctoral degree	69.6	11.86	14.87	64.60	11.95	52.73**	15.72
0 = otherwise	30.4	17.68	18.66	61.73	12.22	44.05**	16.01
**Experience**							
1 = with entrepreneurial experience	27.2	15.99	14.15	68.65**	13.36	52.67	15.72
0 = otherwise	72.8	12.75	16.96	61.88**	11.06	49.13	16.42
**Ownership**							
1 = cofounder	57.6	10.60**	16.26	62.80	12.16	52.20	17.23
0 = otherwise	42.4	17.76**	15.46	64.98	11.91	47.22	14.48
**Funding**							
1 = no external investments	69.6	14.38	17.00	63.99	12.16	49.61	16.56
0 = otherwise	30.4	11.93	14.49	63.12	11.96	51.19	15.67
**Innovation**							
1 = some kind of innovation	79.3	12.81	16.30	63.83	12.26	51.03	16.49
0 = otherwise	20.7	16.81	16.01	63.30	11.46	46.49	15.04
**Total**	100	13.63	16.24	63.72	12.04	50.09	16.23

Note: Significant differences among categories are marked in the Mean columns.

** Significance at the 0.05 level.

**Table 3:** Pearson correlations between independent and dependent variables.

	1	2	3	4	5	6	7	8	9
**1. Age**	1								
**2. Educationy**	0.49***	1							
**3. Experience**	0.16	0.03	1						
**4. Ownership**	0.15	0.20*	0.13	1					
**5. Funding**	-0.18*	-0.13	0.14	-0.38***	1				
**6. Innovation**	0.06	0.07	-0.11	0.21**	-0.28***	1			
**7. Environment**	-0.10	-0.07	0.11	0.02	-0.002	-0.11	1		
**8. Overconfidence**	-0.08	-0.17	0.09	-0.22**	0.07	-0.10	-0.02	1	
**9. Confidence**	0.47***	0.11	0.25**	-0.09	0.03	0.02	-0.15	0.37***	1
**10. Accuracy**	0.43***	0.25**	0.10	0.15	-0.05	0.11	-0.09	-0.73***	0.37***

*Significance at the 0.1 level

** Significance at the 0.05 level

*** Significance at the 0.01 level, (2-tailed)

**Table 4:** Predictors of entrepreneurial overconfidence, confidence and accuracy.

	Overconfidence			Confidence			Accuracy		
	B (SE)	β	Sig.	B	β	Sig.	B	β	Sig.
**Constant**	29.61** (10.67)		0.014	57.19***(6.83)		0.000	30.28*** (9.91)		0.002
**Age**	-0.08 (0.45)	-0.02	0.857	1.38*** (0.29)	0.51	0.000	1.46*** (0.40)		0.001
**Education**	-4.35 (4.26)	-0.12	0.310	-3.18 (2.72)	-0.12	0.246	1.16 (3.95)	0.03	0.769
**Experience**	4.87 (4.02)	0.13	0.230	5.63** (2.58)	0.21	0.032	0.76 (3.74)	0.02	0.839
**Ownership**	-7.24* (3.84)	-0.22	0.063	-4.02 (2.46)	-0.17	0.106	3.23 (3.57)	0.10	0.368
**Funding**	-2.35 (4.18)	-0.07	0.576	0.86 (2.68)	0.03	0.749	3.21 (3.89)	0.09	0.411
**Innovation**	-2.08 (4.42)	-0.05	0.640	1.51 (2.83)	0.05	0.596	3.59 (4.11)	0.09	0.385
**Environment**	-0.23 (0.49)	-0.05	0.640	-0.42 (0.31)	-0.13	0.178	-0.20 (0.45)	-0.04	0.668
**R^2^**	0.09				0.32		0.21		

Standard multiple regression analysis

*Significance at the 0.1 level

** Significance at the 0.05 level

*** Significance at the 0.01 level, (2-tailed)

H1, which assumed that entrepreneurial miscalibration will decrease with age, was initially tested with a Pearson product-moment correlation. Older entrepreneurs were significantly more confident than younger ones, r(90) = 0.472, *p* = 0.000, with age explaining 22% of the variation in the confidence level. The relationship between age and accuracy was also statistically significant, r(90) = 0.430, *p* = 0.000, with age explaining 18% of variation in accuracy. Because these two effects counterbalance one another (i.e., older entrepreneurs were more confident but also more knowledgeable), entrepreneurial age was not associated with the overconfidence score, r(90) = -0.080, *p* = 0.446. We found support for age as a significant determinant of confidence level and accuracy from the multiple regression analysis as well, where the entrepreneurial age coefficient was shown to be positive and significant in the models for confidence (*p* = 0.000) and accuracy (*p* = 0.001).

An independent samples t-test was performed to determine whether the overconfidence score was different for entrepreneurs with Master’s or Doctoral degrees and those with lower education levels, as predicted by H2. As would be expected, the accuracy was statistically significantly different between the two different levels of education, t(90) = -2.425, *p* = 0.017. The percentage of correct answers increased from entrepreneurs with a lower level of education to those with Master’s or Doctoral degrees. The confidence level was not statistically significantly different between the two groups, t(90) = -1.053, *p* = 0.295. The differences in overconfidence between the two groups were also not statistically significant, t(90) = 1.595, *p* = 0.114. If we analyze education as a categorical variable (Master and Doctoral degree are separated into two groups), the relationship between education and confidence and accuracy is monotonic: higher education is related to higher confidence level but also to higher accuracy which counterbalances, thus not leading to overconfidence.

H3, which predicts that entrepreneurial experience negatively affects miscalibration, was tested with an independent samples t-test. The confidence level significantly increased for those entrepreneurs with previous entrepreneurial experience, t(90) = -2.465, *p* = 0.016. Those with previous entrepreneurial experience did not know significantly more than the others, but they thought they did. The results from the bivariate analyses showed that the differences between the two categories regarding their overconfidence score were not statistically significant, t(90) = -0.848, *p* = 0.398. Results from the regression analysis suggested that having entrepreneurial experience in general was associated with a higher confidence level (*p* = 0.032).

An independent samples t-test was also run to determine whether there were differences in overconfidence scores between single entrepreneurs and cofounders (H4). Entrepreneurs who founded their business alone were statistically significantly more overconfident than those with a business partner, t(90) = 2.132, *p* = 0.036. Furthermore, results from the multiple regression analysis showed that ownership structure was a significant determinant of overconfidence among Austrian entrepreneurs: having a business partner was associated with a lower overconfidence score (*p* = 0.063), which provides support for H4. However, the origin of this bias is not obvious, because the difference between these two groups in terms of their confidence level and accuracy was insignificant.

H5 proposed that overprecision is positively related to attracting external investments. The results of the independent samples t-test showed no significant differences in the overconfidence scores between entrepreneurs using external investments and those who did not, t(90) = -0.66, *p* = 0.509, and these results were confirmed by those of the regression analysis.

With respect to H6, entrepreneurs were categorized into two groups according to their innovative activity, as entrepreneurs who perceive their business to be a conventional business model and founders who reported that they applied some kind of innovation, with regard to customers, competition, technology, or some combination of these. No statistically significant difference in the overconfidence scores was observed between these two groups, t(90) = 0.96, *p* = 0.341, a result which was also supported by the results of the regression analysis.

The environmental dynamism scale had a Cronbach's alpha reliability coefficient of 0.61. While this reliability level is not very high, the same scale has been found of acceptable reliability in a study among entrepreneurs from the USA [6]. H7 proposed the existence of a relationship between the degree of self-reported environmental dynamism and overconfidence, and a Pearson correlation was used to test this. No relationship could be found between these two variables, and the results of the multiple regression analysis confirmed this finding.

## 4. Discussion

Considering the high failure rate of newly founded enterprises in Austria, and the proposal in the literature that market success can be predicted by accuracy of calibration [3], this study provides insights on biases in the entrepreneurial context. The study also adds to the entrepreneurial literature in a wider context. The SME sector in Austria has been one of the most resilient ones in Europe during the Eurozone crisis [39]. The vast majority of those engaged in entrepreneurship are motivated by a desire to pursue an opportunity, rather than by necessity [40]. Although opportunity entrepreneurs remain self-employed longer than those pushed to entrepreneurship out of necessity [41], the five-year survival rate in Austria is 50.7% [42]. This approaches the survival rate in Europe, in general, where less than half of enterprises survive for more than a five-year period [43]. In our sample, more than the half of the firms (55%) had been in operation for up to two years, and 76% of the total sample had been in operation up to five years. Most of the respondents (73%) indicated that the current business was their first entrepreneurial experience.

Assessing probability in a well-calibrated way should be taken seriously by those who make decisions in highly uncertain environments when they are not knowledgeable about the probability that different alternative outcomes occur or, better yet, the possible alternative outcomes [44]. Although the research findings have pointed out differences in probabilistic thinking with regard to culture [45], our study corroborated that entrepreneurs from Austria are as overconfident as research findings have shown for their counterparts in the USA. In other words, our study affirmed the existence of a discrepancy between subjective confidence judgments and observed accuracy among entrepreneurs. The entrepreneurs were not perfectly calibrated (i.e. only 71.6% accuracy in questions with 100% confidence level) and these results are related to those reported for probabilistic thinking, i.e., numerical probabilities assigned for general knowledge questions [45]. These findings must be interpreted with caution, as the study was conducted in a European country where information regarding entrepreneurial miscalibration and its determinants is thus far extremely limited.

As suggested in [46], disaggregating the constituent parts of overconfidence bias adds explanatory value, as the overconfidence might originate either from extreme confidence accompanied by insufficient knowledge or a lack of knowledge combined with an inflated perception of knowledge. In accordance with the findings of [47], the more accurate the entrepreneurs are, the more confident they feel about their knowledge. Our findings are also in line with those of [37]: the more confident individuals are, the more overconfident they are.

As previosly stated, a common finding appearing in the miscalibration literature is that answers to hard questions are mostly related to overconfidence, whereas those to easy questions are related to underconfidence. In contrast to previous work, our measurement of entrepreneurial overprecision reflects the hard-easy effect and how the overconfidence score evolves across the different levels of difficulty. We measured overconfidence as suggested by authors of previous studies [24,38,48], and our results indicate that entrepreneurs are most overconfident when answering hard questions and underconfident when answering easy and medium questions. In line with the findings of recent research [38], the overconfidence scores for the easy and hard questions showed significant differences. It would also be informative to examine in detail whether entrepreneurs are prone to higher market entry in industries they perceive as easy compared to those they perceive as difficult. As [4] found in a sample drawn from a university community, entry judgments were related to overplacement or the better-than-average effect, which is greatest in easy tasks unlike the hard-easy effect.

With respect to the determinants of overconfidence and its components, our results show that older entrepreneurs are significantly more confident and more accurate than younger ones. The fact that older respondents are more knowledgeable than the younger ones may suggest that the general-knowledge questions were just more familiar to members of the older generation. Still, this is not an obvious explanation because the questions were not principally related to historical events, as were those in the study of [49], which found that old respondents provided more accurate answers to historical, factual questions than young ones. Entrepreneurial age was not statistically significantly related to overconfidence, and this finding is in line with the results of [26] but conflicts with those of [6,27]. The studies on age-related differences about the accuracy of confidence judgments [50] may provide more valuable insights regarding the differences in probability thinking between young and old entrepreneurs.

The typical approach of measuring overprecision by asking general-knowledge questions was not taken to make a judgment about the general knowledge of the respondents, and they were clearly informed about this in the instructions. Still, highly educated people are expected to provide more accurate answers, as was the case for our sample. If we achieve more heterogeneity on the education level or we separate those with Master’s and Doctoral degrees into two categories, the difference in the confidence level also becomes significant. This provides an affirmative answer to the question asked by [2]: Those who know more, also know more about how much they know.

Previous entrepreneurial experience is a significant predictor of confidence, and those who have it are significantly more confident in their knowledge, but not significantly more accurate. The self-serving attributions, which [51] lists among the most important positive illusions, might play a role here: Individuals attribute positive results to their own decisions and abilities, but ascribe failures to things beyond their control. Future research might address the impact of experience on overconfidence by further differentiating between repeat entrepreneurs who have experienced a business failure and repeat entrepreneurs who have not experienced a business failure [18].

Having a business partner was the only significant predictor of entrepreneurial overconfidence we were able to identify, suggesting that entrepreneurs who founded businesses alone were statistically significantly more overconfident than cofounders. This might even explain their decision to found businesses alone. [11] found that single entrepreneurs were keener to make overestimated forecasts for certain financial results. The relationship between ownership structure and entrepreneurial miscalibration has not been studied yet, so we cannot directly compare our results others. Overconfidence is often ascribed to information-seeking strategies [35,37]. Faced with making a prediction, the individual leans toward one perspective, an act that is facilitated by the mechanisms of associative memory, seeking support for his or her initial judgment. As [35] highlighted, evidence that does not confirm a belief must be sought to form a realistic level of confidence in the belief, and that is why having a business partner that offers counterarguments or simply more options may serve as a cognitive remedy to overconfidence.

Entrepreneurial overconfidence might be of advantage when the founder needs to convince potential investors to make an investment decision [6]. In our sample, we found no statistically significant difference regarding the bias score between those who use and those who do not use external investments. It is also worth noting that less than one-third of entrepreneurs from our sample reported using external investments. Regarding the argument that investors are susceptible to entrepreneurs’ cognitive biases, [28] also found that overconfidence was not associated with funding, but that planning fallacy or being frequently overdue on projects made the difference in fundraising.

Innovation activity, as measured in this study, did not seem to be related to overconfidence in any way. This is unlike the experimental evidence cited by [17], who found that judgmental overconfidence has a negative impact on innovative activity. Measuring innovation on the enterprise level with the use of self-reported innovation rates can be substituted in future research by looking at overconfidence across a range of product introduction decisions (incremental vs. pioneering products) [13].

No significant relationship was found between environmental dynamism and overconfidence. Although we used the same measure for environmental dynamism as [6], this is quite differently perceived by entrepreneurs in the USA and those in Austria (for USA: M = 3.38, SD = 0.72 and for Austria: M = 0.55, SD = 0.92). Considering the same measure and approximately the same sample size, our results show that the sample of Austrian entrepreneurs perceived the environment as more stable than the sample of American entrepreneurs.

As any study in social sciences, also this one is subject to limitations. The sample used in this study is not representative for Austrian entrepreneurs, as young entrepreneurs are over-represented (modal age 31–32), and biased towards male entrepreneurs (only 18.5% of respondents were female). This might be attributed to the gender bias as noted in relevant studies showing that males in general are more likely to respond to web surveys than females (McCabe et al., 2006, in [52]). More than the half (52.2%) of the new sole proprietorships in 2015 in Austria were founded by women [53], which is not comparable to our sample. Note that around three quarters (74.1%) of enterprises born in 2015 in Austria were sole proprietorships [53]. Other than several previous studies, the current study however involved real entrepreneurs to study overconfidence, and not students or potential future entrepreneurs. Additional limitations stem from the selected general knowledge questions, which however were in line with questions used by previous studies, and the way how confidence judgments were elicited–asking for confidence intervals instead of a precise confidence level may yield slightly different results.

## 5. Conclusions

In this study, not all the possible determinants of overconfidence–as reported in the peer-reviewed scientific literature, which is mainly based on research among North-American entrepreneurs–have found to be significant for the Austrian entrepreneurs. The underlying causes for differences in probabilistic thinking and miscalibration between different and similar cultures are still unclear. Scrutinizing how heuristic thinking may be moderated by different factors in different cultures remains a challenge [54].

Studying the determinants of entrepreneurial overconfidence is a challenging milestone towards understanding this cognitive bias, but we should remind ourselves of a comment made by [34] two decades ago: Entrepreneurs who make thoroughly logical assessments face the danger of becoming paralyzed in inaction. Awareness has grown on several issues that are at the core of the overconfidence problem which is viewed as prevalent and, at the same time, has more ruinous potential than other decision biases (Plous, 1993, in [4]). This is particularly important when considering that overconfidence as a bias is associated with the failure of young businesses (those less than five years old), which have been shown to contribute disproportionately to job creation.

It is undeniable that much more can be done to comprehend the overprecision in entrepreneurial context. Various advances have been made towards developing the overprecision measure, which are ready to be tested in entrepreneurial settings. For example, the inclusion of debiasing techniques, such as providing feedback or teaching the subjects about the concept of calibration may improve our comprehension of entrepreneurial overconfidence [24,36]. Being aware alone of the fallibility in one’s judgment can count for more than many other cognitive remedies to overconfidence [35]. Other possible pathways for future research would be to consider running a field experiment like [55] did to study excess market entry, or applying an incentive-compatible method to measure overprecision like the one developed and tested among students by [56]. Future research can also examine in more detail whether the factors are differentially associated to overconfidence across different levels of difficulty.

## Supporting information

S1 File Questionnaire and categorization of questions. This file contains the used questionnaire and describes how the general knowledge questions were categorized into easy, medium and hard questions.
(PDF)

S2 File Survey data. This file contains the raw data collected in the survey.
(XLSX)
