# Supplementary material for: “Yes, we know!” (Over)confidence in general knowledge among Austrian entrepreneurs
Source: PLoS One. 2018 May 8;13(5):e0197085. doi: 10.1371/journal.pone.0197085 (PMC5940190; doi:10.1371/journal.pone.0197085)
Supplement: S1 File — This file contains the used questionnaire and describes how the general knowledge questions were categorized into easy, medium and hard questions. (PDF) [file pone.0197085.s001.pdf]

Supporting Information S1

Supplement S1 A: Questionnaire

The following questionnaire was used in the study, accompanied by a short introductory text. Note that the original questionnaire was in German (presumably the native language of most respondents). Here we provide a translation of the original questionnaire. Note that questions 5-17 were only asked if the respective pre-conditions were being met. Respondents who had never founded a business and are not in the process of planning to do so exited the questionnaire after question 4.

Part 1: Questions regarding the entrepreneur and their business

1. Age

2. Gender [male, female]

3. Highest completed degree [PhD, Master; Bachelor; A-levels; mandatory school]

4. How much experience do you have with founding a business? [I have (co-)founded one enterprise; I have (co-) founded several enterprises; I am currently preparing to found one enterprise; I have not yet founded and enterprise and do not plan to do so at the moment]

5. [*conditional*] How many enterprises did you found prior to your current enterprise? [one; two; three or more]

6. [*conditional*] In which year was your current enterprise founded?

7. [*conditional*] Did you found the enterprise alone or together with (a) partner(s)? [alone; together with partner(s)]

8. [*conditional*] Are you planning to found the enterprise alone or together with (a) partner(s)? [alone; together with partner(s)]

9. [*conditional*] Are you using external investments*? [yes; no]

* Venture Capitalists, Business Angels, or other external investors who are not part of the founding team.

10. [*conditional*] Do your costumers perceive your product/service as something new? [All customers; most costumers; some customers; few customers; no customers]

11. [*conditional*] How many providers of comparable products or services are available to your potential customers? [many competitors, few competitors, no competitors]

12. [*conditional*] Did technologies or processes for this product / service exist three years ago? [Yes; no]

13. [*conditional*] The competitive environment for my product / service is unpredictable. [5-level Likert scale; 1 = fully disagree; 5 = fully agree]

14. [*conditional*] The competitive environment for my product / service is hostile. [5-level Likert scale; 1 = fully disagree; 5 = fully agree]

15. [*conditional*] My branch of industry is changing constantly. [5-level Likert scale; 1 = fully disagree; 5 = fully agree]

16. [*conditional*] There is a high number of market entries and exits of enterprises in my branch of industry. [5-level Likert scale; 1 = fully disagree; 5 = fully agree]

17. [*conditional*] This branch of industry is going through many technological and regulatory shocks. [5-level Likert scale; 1 = fully disagree; 5 = fully agree]

Part 2 – General knowledge questions

In part two, the respondents were asked to (1) answer to general knowledge questions (single-choice from three offered answers) and (2) to indicate their confidence level on a scale from 33% (guess) to 100% (absolutely sure). Correct answers are marked in bold letters here.

Q1. How is an instant camera also called? [Canon camera; **Polaroid camera**; Minolta camera]

Q2. Where are flounders mainly to be found? [In coral reefs; **at the bottom of the sea**; in common reed]

Q3. Which sauce is traditionally served with Thanksgiving turkey in the USA? [Blueberry sauce; red currant sauce; **cranberry sauce**]

Q4. Where does the Nobel Prize winner in Literature, Gabriel Garcia Marquez, come from? [**Colombia**; Spain; Venezuela]

Q5. What artistic movement does Anacreontics belong to? [**Rococo**; Romanticism; Realism]

Q6. What is the name of a spicy chili sauce? [**Tabasco**; Curacao; Macao]

Q7. How many letters are there in the Russian alphabet? [40, **33**, 26]

Q8. Tosca is an opera by...? [**G. Puccini**, G. Verdi, A. Vivaldi]

Q9. What is the name of the Greek Goddess of Wisdom? [**Pallas Athena**, Nike, Penelope]

Q10. What is the most abundant metal in Earth's crust? [Iron, **aluminum**, copper]

Q11. What is the word for an "uninformed person"? [Ignatius, **ignorant**, ideologue]

Q12. Who was the first person to fly around the Eiffel tower in an airship? [**Santos-Dumont**; Count Zeppelin, Saint-Exupéry]

Q13. How is the snow house of Eskimos called? [Wigwam; **Iglu**; Tipi]

Q14. Which enterprise was co-founded by Bill Gates? [Intel; **Microsoft**; Dell Computers]

Q15. How is the fastening month in Islam called? [Sharia; **Ramadan**; Imam]

Q16. What language does the term "Fata morgana" come from? [**Italian**, Arabic, Swahili]

Q17. What do camels store in their humps? [**Fat**, water, milk]

Q18. What is ascorbic acid? [Apple vinegar, **vitamin C**, vitamin A]

Supplement S1 B: Categorization of questions

Based on the mean accuracy of the respondents (n=92), the questions were categorized into easy (70-100% accuracy), medium (40-70% accuracy) and hard (0-40% accuracy) questions. Table B1 presents the categorization and mean accuracy in answers to the general knowledge questions.

**Easy (70-100%)**	Q3= 88%<br>Which sauce is traditionally served with Thanksgiving turkey in the USA?<br>(blueberry sauce, red currant sauce, **cranberry sauce**)	Q11=81.5%<br>What is the word for an "uninformed person"?<br>(ignatius, **ignorant**, ideologue)	Q18=77.2%<br>What is ascorbic acid?<br>(apple vinegar, **vitamin C**, vitamin A)	Q9=77.2%<br>What is the name of the Greek Goddess of Wisdom?<br>(**Pallas Athena**, Nike, Penelope)
**Medium (40-70%)**	Q7= 59.8%<br>How many letters are there in the Russian alphabet?<br>(40, **33**, 26)	Q8=50%<br>Tosca is an opera by...?<br>(**G. Puccini,** G. Verdi, A. Vivaldi)	Q4=47.8%<br>Where does the Nobel Prize winner in Literature, Gabriel Garcia Marquez, come from?<br>(**Colombia,** Spain, Venezuela)	Q5=40.2%<br>What artistic movement does Anacreontics belong to?<br>(**Rococo,** Romanticism, Realism)
**Hard (0-40%)**	Q17=35.9%<br>What do camels store in their humps?<br>(**fat**, water, milk)	Q12=27.2%<br>Who was the first person to fly around the Eiffel tower in an airship?<br>(**Santos-Dumont,** Count Zeppelin, Saint-Exupéry)	Q10=9.8%<br>What is the most abundant metal in Earth's crust?<br>(iron, **aluminum,** copper)	Q16=6.5%<br>What language does the term "Fata morgana" come from?<br>(**Italian**, Arabic, Swahili)

S1 Table: Level of accuracy in answers to general knowledge questions in the sample of Austrian entrepreneurs (n=92), categorized into easy, medium and hard questions.
